# Frequent detection of IFN-gamma -producing memory effector and effector T cells in patients with progressive multifocal leukoencephalopathy

**DOI:** 10.3389/fimmu.2024.1416074

**Published:** 2024-07-17

**Authors:** Marie-Ghislaine de Goër de Herve, Manon Dekeyser, Houria Hendel-Chavez, Elisabeth Maillart, Céline Labeyrie, David Adams, Thibault Moreau, Catherine Lubetzki, Caroline Papeix, Bruno Stankoff, Jacques Gasnault, Yassine Taoufik

**Affiliations:** ^1^ INSERM 1186, Institut Gustave Roussy, Université Paris-Saclay, Villejuif, France; ^2^ Department of Neurology, Hôpital Pitié-Salpêtrière, Assistance Publique - Hôpitaux de Paris, Paris, France; ^3^ Department of Neurology, Hôpital Bicêtre, Assistance Publique - Hôpitaux de Paris, Le Kremlin-Bicêtre, France; ^4^ Department of Neurology, CHU Dijon, Dijon, France; ^5^ Department of Neurology, Hôpital Tenon, Assistance Publique - Hôpitaux de Paris, Paris, France; ^6^ Unité de Suite et Réadaptation, Department of Internal Medicine, Hôpital de Bicêtre, Assistance Publique - Hôpitaux de Paris, Le Kremlin-Bicêtre, France

**Keywords:** JC virus, progressive multifocal leukoencephalopathy, AIDS, multiple sclerosis, natalizumab, effector memory T cells, effector T cells, IFN-γ release assay

## Abstract

**Introduction:**

Progressive Multifocal Leukoencephalopathy (PML) is a rare and deadly demyelinating disease caused by JC virus (JCV) replication in the central nervous system. PML occurs exclusively in patients with severe underlying immune deficiencies, including AIDS and hematological malignancies. PML has also emerged as a significant threat to patients on potent new immunosuppressive biologics, including natalizumab in multiple sclerosis.

**Methods:**

Here, we developed an IFN-γ release assay (IGRA) that mainly detects JCV-specific effector memory T cells and effectors T cells in the blood.

**Results:**

This assay was frequently positive in patients with active PML (with a positive JCV PCR in CSF) of various underlying immunosuppression causes (84% sensitivity). Only 3% of healthy donors had a positive response (97% specificity). The frequency of positivity also increased in multiple sclerosis patients according to the time on natalizumab (up to 36% in patients treated for more than 48 months, who are considered at a higher risk of PML).

**Discussion:**

The results show this assay’s frequent or increased positivity in patients with PML or an increased risk of PML, respectively. The assay may help to stratify the risk of PML.

## Introduction

Progressive multifocal leukoencephalopathy (PML) is a rare but deadly demyelinating disease. It is caused by human JC polyomavirus (JCV) replication in oligodendrocytes and astrocytes, and almost exclusively affects immunosuppressed patients (1–8). JCV is a ubiquitous virus that asymptomatically infects a large proportion of the adult population worldwide (1–8). Various situations associated with severe cellular immunodeficiency allow JCV to replicate in the central nervous system (CNS), leading to PML (1–8). Until recently, these situations consisted mainly of AIDS and, to a lesser extent, hematological malignancies or chronic inflammatory diseases and transplantation due to the heavy therapeutic immunosuppression (1–10). The incidence and severity of PML vary according to the predisposing underlying disease (10). The overall incidence has been estimated at 0.5 per 100,000 person-years in the USA (10), 0.11 per 100,000 person-years in France (9) and 0.029 per 100,000 person-years in Japan (11). The overall mortality at one year was 38.2% in France (9).

Recently, PML has emerged as a severe adverse effect of potent new immunosuppressive biotherapies used to treat autoimmune diseases, allogeneic graft rejection, or hematological malignancies, such as rituximab, natalizumab, efalizumab, belatacept, alemtuzumab or daratumumab, and other immunomodulatory agents such as belatacept, dimethyl fumarate, ocrelizumab and fingolimod used in multiple sclerosis (1, 2, 12–24). Natalizumab, an anti-α4 integrin that blocks T-cell trafficking through the blood-brain barrier, has proven effective in relapsing-remitting multiple sclerosis (25–27). Still, the risk of PML limits its long-term use (2, 5, 13, 14, 16, 24, 28, 29). Identification of patients at risk of developing PML is thus an important challenge. Detection and quantification of anti-JCV antibodies in sera of patients on natalizumab represented a significant advance in the strategies to stratify the risk of PML in patients on natalizumab (30–33). A high level of JCV antibodies may indicate sustained virus activity and an increased risk of PML in patients on natalizumab (34). Immunological control of JCV infection depends primarily on the T-cell immune response. Detection of JCV-specific T cells with effector functions (effector and effector memory T cells) may point to ongoing JCV replication in extra-renal sites (35). Those T cells rapidly release cytokines such as gamma interferon (IFN-γ) when re-exposed to antigen (35, 36). Here, we developed a whole blood-specific IFN-γ release assay to detect effector/memory effector JCV-specific T-cell response. We tested it in patients with PML or at risk of developing PML.

## Patients and methods

### Patients

This study involved 33 healthy donors and 110 patients, including 67 patients with remitting-relapsing MS. Patients with MS were enrolled in the neurology departments of Pitié-Salpêtrière, Tenon and Bicêtre hospitals (Paris, France). Some MS patients on natalizumab were tested at 2 or 3 time points (a total of 98 samples were tested). The interval between samples ranged from 1 to 14 months. At the time of sampling, 57 MS patients had been on natalizumab for 2 to 82 months, and 10 MS patients had stopped receiving natalizumab 1 to 29 months previously. The study also involved 19 patients with recent-onset PML (less than 1 year from who had evidence of JCV replication in the CNS (positive JCV PCR in the CSF) (active PML) and 12 PML survivors with no evidence of CNS JCV replication (negative JCV PCR in the CSF). PML was diagnosed on the basis of clinical and virological findings and magnetic resonance imaging (MRI), as previously described (37). The PML patients had a variety of immunodeficiencies including AIDS, natalizumab treatment, sarcoidosis, lymphoma/leukemia, or allogeneic bone marrow transplantation. The patients with active PML had been diagnosed less than 1 year before sampling [median: 3.5 months; range: 0.4 -11.2 months] and all were PCR-positive for JCV in CSF. All the PML survivors had been diagnosed more than 1 year previously (median: 53.15 months; range: 14.3 to 188 months) and were PCR-negative for JCV in CSF at the time of blood sampling. Blood samples from PML patients were obtained in several French clinical centers (USR, Department of Internal medicine, Bicêtre Hospital; Departments of Neurology of Dijon, Tenon and Saint-Antoine Hospitals). Controls were 12 AIDS patients with neurological diseases other than PML, including cerebral toxoplasmosis, HIV encephalitis and CNS lymphoma, who were recruited at the USR, Department of Internal Medicine, Bicêtre Hospital. Samples from 33 healthy donors were also tested. Written informed consent was obtained from each patient (or next-of-kin if decision-making was impaired) and each healthy donor. The study was approved by the CPP IDF VII ethics committee (Comité pour la Protection des Personnes Ile-de-France VII, Pitié-Salpêtrière hospital, France).

### IFN-γ release assay

A pool of 46 peptides, each composed of 25 amino acids (0.25 μg/mL each), overlapping by 10 amino acids and spanning the entire VP1 and VP2 proteins of JCV (American Peptide Company, Sunnyvale, CA), was added to 1 mL of whole blood for 16 h at 37°C. A positive control (activation with PHA 0.5 µg/mL) and a negative control were used. After peptide activation, plasma was harvested and IFN-γ release was measured by ELISA (Qiagen, Gaithersburg, MD, USA). The results are provided in IU/mL [one IU approximately corresponds to 40 pg/mL (38)].

### Sorting of T cell subsets

CD4+ T cells were enriched from PBMC by using magnetic beads (CD4 Microbeads, Miltenyi Biotec). CD4-enriched and CD4-depleted cells (containing CD8+ T cells) were stained with the following antibodies: CD3-FITC, CD8-Alexa Fluor700, CD4-V500, CCR7-PE-CF594, CD95-APC, CD62L-V450 (BD Biosciences), CD45RO-PE-Vio770, CD45RA-PerCP-Vio700, CD27-APC-Vio770 (Miltenyi Biotec). Central memory (CD45RA-, CD45RO+, CCR7+, CD62L+, CD27+), effector memory (CD45RA-, CD45RO+, CCR7-, CD62L-, CD27+), stem cell memory (CD45RA+, CD45RO-, CCR7+, CD62L+, CD27+, CD95+), and naive CD4+ and CD8 T cells (CD45RA+, CD45RO-, CD62L+, CCR7+, CD27+, CD95-) were then sorted from CD4-enriched and CD4-depleted PMBC (BD FacsAria). Non-T cells were also sorted (CD3-negative cells within the lymphocyte gate) for coculture experiments.

### Cell activation and detection of IFN-γ production by flow cytometry

Sorted CD4+ or CD8+ T cells were cocultured for 16 hours with non-T cells in the presence of the VP1 and VP2 peptide pool (0.25 µg/ml each peptide). The cells were washed, stained with the same antibodies as those used for cell sorting, then fixed and permeabilized (BD Cytofix/Cytoperm, BD Biosciences) before staining for intracellular IFN-γ (anti-IFN-γ PE, clone B27, BD Biosciences) and flow cytometric analysis (BD LSR Fortessa). Data were analyzed with FlowJo software.

In some experiments, PBMC were directly activated with JCV peptides then stained with anti-CD3, anti-CD4, anti-CD8, anti-CD45RO, anti-CD45RA, anti-CCR7 and for intracellular IFN-γ production (same antibodies as above) prior to flow cytometry.

### Statistical analysis

Data were analyzed with Prism software (GraphPad). Differences between groups were analyzed using the Mann-Whitney test for unpaired continuous variables. Chi-square test was used for categorical variables and the Spearman rank test for correlation studies.

## Results

### Study design

This work aimed to develop a whole-blood JCV-specific IFN-γ release assay (see Methods) following activation for a short period, 16 h, with a pool of overlapping peptides spanning the entire JCV proteins VP1 and VP2. IFN-γ released by T cells was measured in the plasma. The assay was tested in patients with relapsing-remitting multiple sclerosis (MS) who had been on natalizumab for various periods; patients with recent-onset PML (active PML <1 year since diagnosis and PCR positivity for JCV in CSF at the time of sampling); and PML survivors (“inactive PML”: >1 year since diagnosis, PCR-positive for JCV in CSF at diagnosis but negative at the time of sampling). PML patients had various underlying immunosuppressive disorders (HIV infection, treatment with natalizumab, lymphoma, or sarcoidosis). As HIV infection was a frequent cause of immunosuppression among PML patients, we also tested a control group of patients with AIDS-related opportunistic neurological diseases other than PML (cerebral toxoplasmosis, HIV encephalitis, and CNS lymphoma). CD4 T cell counts in the latter patients were similar to those in the AIDS patients with active PML, suggesting a similar degree of immunodeficiency (195 cells/mm3 [28–979] in active PML, 201 cells/mm3 [24–898] in neuro-AIDS; median [range], p=0.746, Mann-Whitney test).

### T_EM_ and T_EMRA_ cells accounted for the bulk of IFN-γ-producing CD4 and CD8 T cells after short activation with JCV peptides

**Figure 1:**
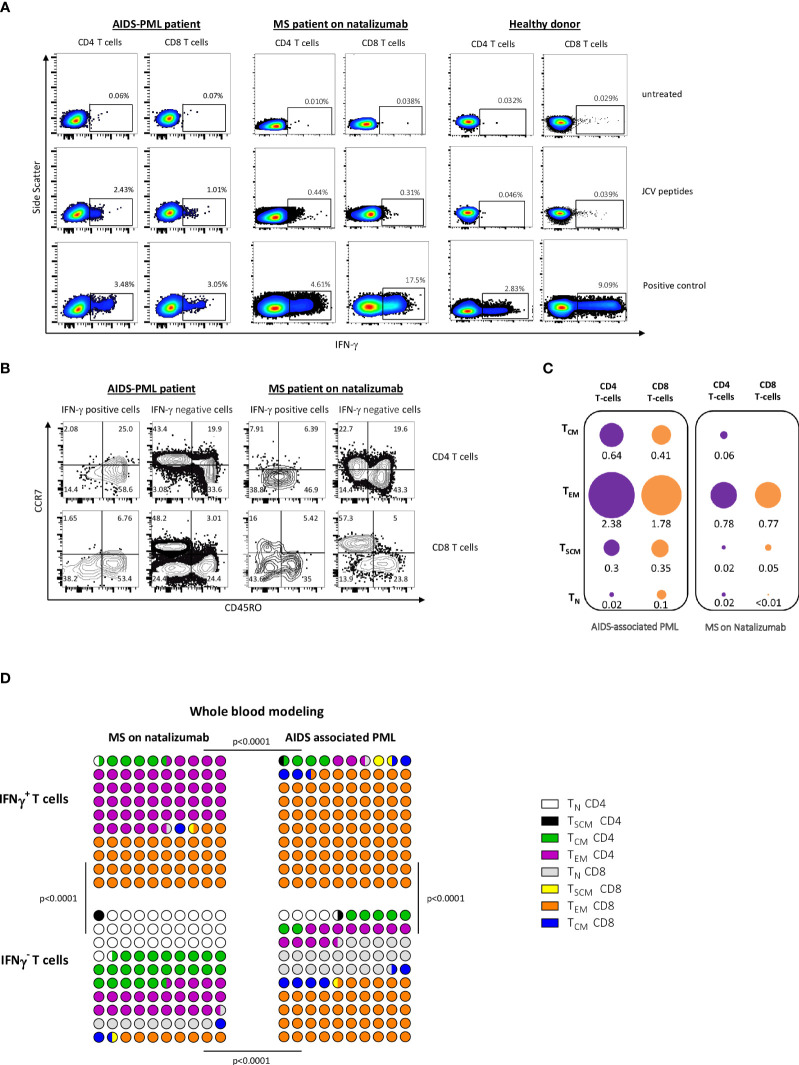
After short-term PBMC activation with JCV peptides, T_EM_ and T_EMRA_ are the main IFN-γ-producing cell subset. **(A, B)** PBMC from an MS patient on natalizumab were activated with JCV peptides for 16 (h) In **(A)** Intracellular IFN-γ production by CD4 and CD8 T cells, gated among PBMCs, was analyzed by flow cytometry. Panel **(B)** shows the phenotype of IFN-γ-producing and non-producing cells among CD4+ T cells (top) and CD8+ T cells (bottom). **(C, D)** Naive (T_N_), stem cell memory (T_SCM_), central memory (T_CM_), and effector memory (T_EM_) CD4 and CD8 T cells were sorted from PBMC from patients with AIDS-associated PML and PML-free MS patients on natalizumab. Terminally differentiated effector T cells (T_EMRA_) which are highly susceptible to apoptosis could not be tested. Those highly purified cells were activated with JCV peptides for 16 h in the presence of T-cell-depleted autologous PBMC. Intracellular IFN-γ production by CD4 and CD8 T cells was analyzed by flow cytometry. In **(C)**, the mean percentages of IFN-γ positive cells within each sorted CD4+ or CD8+ T cell subset are shown. The surface areas of ​​the discs are proportional to the percentages of IFN-γ-positive cells in each sorted T cell subset. The area corresponding to the percentage of IFN-γ cells in CD8 T_EM_ in AIDS-associated PML patients was determined arbitrarily. The other surfaces were determined proportionally. Panel **(D)** represents the importance of each subset in 100 IFN-γ producing CD4 and CD8 sorted T cells from MS patients (left) and patients with AIDS-associated PML (right). Results were calculated from the mean percentage of IFN-γ-producing cells in each sorted CD4 or CD8 T-cell subset (see **C**) and the mean absolute number of each CD4 or CD8 T-cell subset in 1 mm^3^ of blood. The same procedure has been performed for IFN-γ negative cells in each sorted CD4 or CD8 T-cell subset. Statistical analysis was performed using a Chi-square test. In **(C, D)**, means were calculated from values obtained in 6 PML patients and 4 MS patients with positive IFN-γ response after activation with JCV peptides.

We first analyzed the phenotype of IFN-γ producing cells after a short-term activation (16h) with VP1 and VP2 peptides. As shown in **Figure 1A**, the peptides activated both JCV-specific CD4 and CD8 T cells. The majority of IFN-γ-producing T cells had a CD45RO+ CCR7- or a CD45RO- CCR7- phenotype (**Figure 1B**). Those phenotypes are characteristic of effector memory T cells (T_EM_), and terminally differentiated effector T cells (called T_EFF_ or T_EMRA_), respectively (39). Most T_EM_ and T_EMRA_ were also CD45RA - and CD45RA+, respectively (not shown). *In vivo*, specific T_EM_ and T_EMRA_ may decline once the cognate antigen has been cleared, and their significant presence may, therefore, point to ongoing pathogen replication (35, 40–42). T_EM_/T_EMRA_ rapidly release cytokines such as IFN-γ when re-exposed to antigen (35, 40–42). By contrast, long-term antigen activation for several days may allow long-term quiescent T cell memory cells (mainly central memory T cells) to be reactivated and expand (35, 40–42), meaning that a positive response following prolonged antigen activation does not necessarily signify an ongoing immune response.

To rule out that a short-term activation may have induced differentiation to T_EM_ or T_EMRA_ from less differentiated memory T cells, we sorted CD4+ and CD8+ stem cell memory T cells (T_SCM_), central memory T cells (T_CM_), and effector memory T cells (T_EM_) from AIDS-associated PML patients and MS patients on natalizumab (see Methods). Healthy donors were not tested as most of them had no reactivity in terms of IFN-γ production following short-term activation with JCV peptides (35). Naive T cells were sorted as controls. Because of a high rate of cell death following sorting, terminally differentiated effector T cells could not be tested (T_EMRA_ are highly susceptible to apoptosis (43)). Sorted naive and memory T-cell subsets mixed with T-cell-depleted PBMC were activated with JCV peptides for 16 h. Intracellular IFN-γ was analyzed by flow cytometry. **Figure 1C** shows the percentages of IFN-γ-producing cells among each T-cell subset. **Figure 1D** summarizes the contribution of each sorted naive and memory subset to the pool of IFN-γ-producing T cells after JCV peptide activation. The modeling considered the percentage of IFN-γ producing cells among each cell subset and the abundance of each cell subset in whole blood (see legends). In both AIDS-related PML patients and MS patients, the main T cell subset that produced IFN-γ in response to JCV peptides was the T_EM_ subset (**Figure 1D**). In AIDS–related PML patients, the ratio of CD4 T_EM_ to CD8 T_EM_ among IFN-γ-producing T cells was very low (ratio of 0.03 in our whole-blood model in **Figure 1D**). This ratio was much higher in MS patients on natalizumab (1.18) (**Figure 1D**). The overrepresentation of CD8 T cells among IFN-γ-producing T_EM_ in AIDS-related PML was due to the CD4 lymphopenia. Indeed, as shown in **Figure 1C**, similar percentages of CD4 and CD8 T cells produced IFN-γ in response to JCV peptides in AIDS-related PML patients. As shown in **Figure 1D**, naive cells contributed significantly to the pool of IFN-γ non-producing T cells after JCV peptide activation (40% for MS patients on natalizumab and 28.5% for AIDS-PML patients).

### Frequent positivity of the JCV-specific IGRA test in patients with active PML

**Figure 2:**
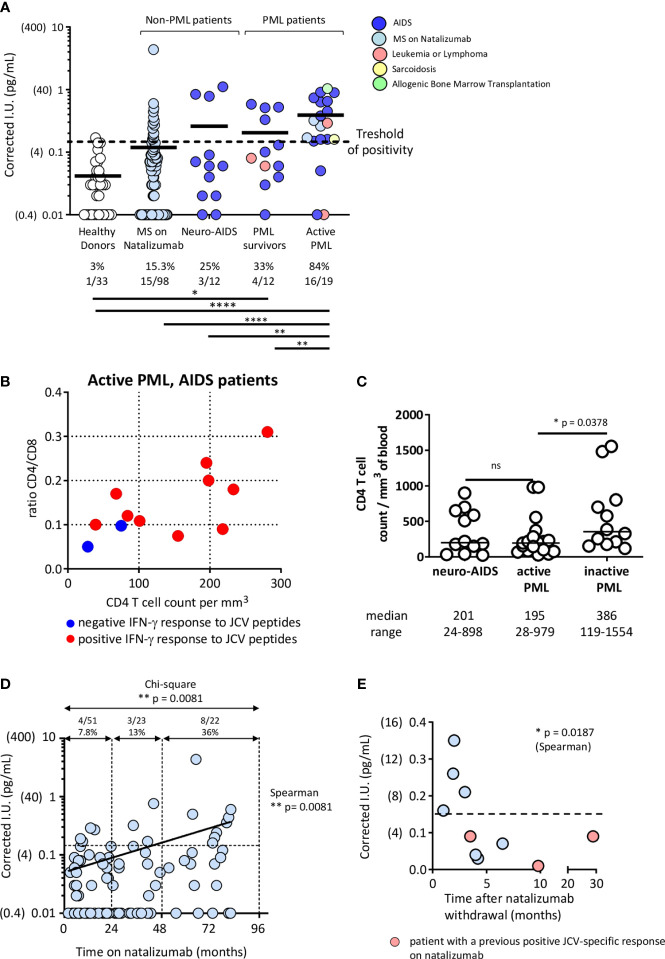
Patients with PML or a high PML risk have a positive JCV IFN-γ release test. **(A)** Healthy donors, MS patients on natalizumab for various times, neuro-AIDS patients without PML, and PML patients with various underlying immunosuppressive conditions were tested in the IFN-γ release assay described in Methods. PML patients were divided into PML survivors (> one year from diagnosis and no evidence of ongoing JCV replication in the CNS, based on a JCV PCR in CSF that became negative) and patients with active PML (< one year since diagnosis and evidence of virus replication in the CNS, based on a positive JCV PCR in CSF). The numbers and percentages of test-positive healthy donors or patients in each group are indicated. IFN-γ production values obtained in peptide-activated blood tubes are corrected by subtracting the values obtained in non-activated blood tubes. The positivity cutoff was 0.15 (corresponding to the mean + 2 SD of values obtained in non-activated blood tubes in 33 healthy donors). Results were analyzed with Chi-square test. Significances are indicated: * *p*<0.05; ** *p*<0.01; **** *p*<0.0001. The values obtained in non-activated tubes did not vary significantly between the groups of healthy donors and patients (Healthy donors: 0.07 [0.04] (mean [SD]); Multiple Sclerosis patients: 0.09 (0.06); Neuro-AIDS patients: 0.08 (0.04); Active PML patients: 0.09 (0.04); PML survivors: 0.06 (0.02) and active PML patients: 0.09 (0.04)). Panel **(B)** shows CD4 T cell counts and CD4/CD8 T cell ratios in AIDS-related PML patients according to the results of the IFN-γ release assay. Panel **(C)** shows the CD4 T cell counts in the different groups of AIDS patients. The CD4 T cell count was not measured in our group of healthy donors. However, a previous representative study has shown that in HIV-seronegative adult patients, the CD4 cell count ranges between 582 and 2628 cells/mm^3^ (median: 1119 cells/mm^3^) (44). Results were analyzed with Mann-Whitney test. In **(D)**, the time on natalizumab by MS patients shown in **(A)** was plotted against corrected IFN γ values. The Spearman rank test was used to analyze the correlation. The Chi-square test was used to analyze the rate of IFN-γ positivity after 0-24 months, 24-48 months, and >48 months on natalizumab. Panel **(E)** plots the results of the IFN-γ release assay against the time since natalizumab withdrawal among MS patients who had stopped receiving natalizumab. Pink circles represent patients who previously tested positive for IFN-γ release. The Spearman rank test was used to analyze the correlation.

We then tested our IFN-γ assay on whole blood. The results of the IFN-γ release assay were expressed as delta IFN-γ international units (ΔIU = result (IU) in the activated tube – result (IU) in the non-activated tube). The positivity cutoff was set at 0.15, corresponding to the mean IU obtained for negative controls (untreated blood tubes) from healthy donors + 2 SD. The ΔIU of positive controls (blood tubes activated with PHA) ranged from 2 to >10 in the patients and healthy controls. As shown in **Figure 2A**, one of the 33 healthy donors (3%) had a positive IFN-γ response to JCV peptides. Sixteen of the 19 active PML patients (84%) responded positively. Two negative patients had AIDS-associated PML and were among the AIDS patients with the lowest CD4 T cell counts and CD4/CD8 ratios. Those two patients died within 3 months after the blood testing (**Figure 2B**). The third negative patient had lymphoma treated with rituximab (216 CD4 T cells and 196 CD8 T cells per mm^3^ but no B cells). The peptides (25 amino acids) used in the assay may be substantially presented by B cells (that express both MHC class II and MHC class I molecules) to CD4 T cells and CD8 T cells, respectively. CD4 T cells represent a significant fraction of cells that produce IFN-γ in response to JCV peptides (**Figures 1A–D**), and both CD4 T cells and B cells (45) may also provide helping signals for CD8 T cell functionality. The virtual absence of CD4 T cells in some AIDS patients or the loss of B cells in patients on rituximab may, therefore, impact the test. Only 33% of patients with inactive PML (PML survivors) had a positive response. Survivors of AIDS-associated PML had higher CD4 cell counts than patients with active PML, indicating some degree of immune recovery on antiretroviral treatment (**Figure 2C**). The AIDS patients with neurological diseases other than PML were positive in 25% of cases despite a similar degree of immune deficiency, based on their CD4 T cell counts, to that of patients with active PML (**Figure 2C**). None of these neuro-AIDS patients and none of the patients with inactive PML were PCR-positive for JCV in CSF at the time of testing. In contrast, all the patients with active PML were positive. Thus, active JCV replication in the brain was associated with a positive response in the IFN-γ release assay.

### The frequency of positivity also increased in MS patients according to the time on natalizumab

Among the patients with multiple sclerosis treated with natalizumab, the assay was positive in 15.3% of the samples tested (**Figure 2A**). The risk of PML is known to increase with time on natalizumab (7, 8). We found a positive correlation between the time on natalizumab and positivity in our IFN-γ release assay (**Figure 2D**). Among patients who had been on natalizumab for less than 24 months, the proportion of those with positive IFN-γ responses was similar to that of the healthy donors (7.8% and 3%, respectively, p=0.36, Chi-square test). Interestingly, the proportion was 13% among patients who had been on natalizumab for 24 to 48 months and 36% among patients who had been on natalizumab for more than 48 months (**Figure 2D**). Among patients who had stopped taking natalizumab, we found a negative relationship between IFN-γ positivity and the time since drug withdrawal (**Figure 2E**). Thus, among MS patients, positivity in the IFN-γ release assay tends to become more likely with the time on natalizumab and, thus, with the risk of PML.

## Discussion

Our results suggest that PML, and therefore active JCV replication in the CNS based on a positive JCV PCR in CSF, is strongly associated with the positivity of our JCV-specific IGRA test, which detects the presence of JCV-specific T_EM_ and T_EMRA_ in peripheral blood. The detection frequency of those cells decreased in PML survivors who cleared JCV from CSF. The observed anti-JCV T cell response may not necessarily be able to control JCV replication in the CNS. Indeed, there are several examples of chronic viral infections, including HIV infection, in which specific CD4 and CD8 T cells have lost several critical antiviral functions, including cytotoxicity, but remain able to produce IFN-γ (46). Several mechanisms may be possibly involved in this poor functionality, including a lack of CD4 help, overexpression of inhibitory receptors, T cell anergy, or increased Treg responses (19, 46–50). The immune recovery that occurs in AIDS patients on effective ART, following the suppression of HIV replication, may improve the functionality of anti-JCV T_EM_ and T_EMRA_, enabling them to control virus replication in the CNS. This virus clearance and, therefore, the decrease of antigen activation by dendritic cells migrating from the brain may lead to a drop of JCV-specific T_EM_ and T_EMRA_ in the blood that may become undetectable by the JCV-specific IGRA test.

We also found that the positivity of the JCV-specific IGRA increased in MS patients with the time on natalizumab, which correlates with the risk of PML. In MS patients, while there is no evidence that natalizumab impairs JCV-specific T-cell functions, this therapeutic monoclonal antibody prevents T-cell trafficking to the brain through the blood-brain barrier, creating local CNS immunosuppression. Detecting JCV-specific T_EM_ and T_EFF_ cells in some patients’ blood on natalizumab suggests two points.

Firstly, direct and indirect evidence of ongoing JCV replication in the CNS of some patients on natalizumab has already been reported (16, 35, 51, 52). JCV replication might initially be intermittent and low, subsequently intensifying progressively to cause substantial demyelination and lead to PML. We have previously shown that the presence of JCV-specific T_EM_ in the blood is unrelated to JCV replication in renal epithelial cells, which is responsible for the viruria frequently observed in the general population (35). By contrast, a high proportion of PML patients with a positive JCV PCR in CSF showed detectable anti-JCV T_EM_ and T_EFF_ in blood. The frequency of detection of those cells decreased in AIDS-associated PML survivors who cleared JCV from CSF following effective ART treatment.

Secondly, the increased positivity of the JCV-specific IGRA test in MS patients on prolonged natalizumab suggests that this therapeutic monoclonal antibody does not prevent peripheral T cell activation by JCV antigens present in the CNS. Further investigations are necessary to determine the impact of natalizumab on antigen-presenting-cell trafficking from the brain to peripheral locations such as brain-draining cervical lymph nodes, which may involve newly identified CNS lymphatic vessels (53). In PML patients, the influx of activated T cells following natalizumab withdrawal and plasma exchange triggers an immune reconstitution inflammatory syndrome (IRIS) (29, 54, 55). Cells that enter the CNS are likely to include JCV-specific T cells. Our finding that JCV-specific effector memory T cells accumulate in the blood of natalizumab-treated MS patients who have PML or are at a high risk of PML supports the involvement of JCV-specific T cells in this inflammatory syndrome.

Together, our results suggest that the positivity of the JCV-specific IGRA test correlates with virus replication in the CNS, as shown in patients with active PML patients, and may suggest some levels of virus replication in patients on prolonged natalizumab that may lead to PML. Therefore, this assay may help in the current strategies to better mitigate the risk of PML in patients on natalizumab and possibly on other immunosuppressive biotherapies.

## Data availability statement

The original contributions presented in the study are included in the article/supplementary materials, further inquiries can be directed to the corresponding author/s.

## Ethics statement

The studies involving humans were approved by Comité de Protection des Personnes Ile-de-France VII (Le Kremlin-Bicêtre). The studies were conducted in accordance with the local legislation and institutional requirements. Written informed consent was obtained from each patient (or nextof-kin if decision-making was impaired) and each healthy donor.

## Author contributions

M-GGH: Conceptualization, Formal analysis, Methodology, Resources, Supervision, Visualization, Writing – original draft, Writing – review & editing. MD: Supervision, Validation, Visualization, Writing – original draft, Writing – review & editing. HH-C: Conceptualization, Data curation, Formal analysis, Investigation, Validation, Writing – original draft. EM: Resources, Writing – original draft. CLa: Resources, Writing – original draft. DA: Resources, Writing – original draft. TM: Resources, Writing – original draft. CLu: Resources, Writing – original draft. CP: Resources, Writing – original draft. BS: Resources, Writing – original draft. JG: Formal analysis, Investigation, Methodology, Resources, Validation, Visualization, Writing – original draft, Writing – review & editing. YT: Conceptualization, Formal analysis, Funding acquisition, Methodology, Project administration, Supervision, Validation, Visualization, Writing – original draft, Writing – review & editing.
